# Multiparametric MRI evaluation of bone sarcomas in children

**DOI:** 10.1186/s13244-022-01177-9

**Published:** 2022-03-01

**Authors:** Emilio J. Inarejos Clemente, Oscar M. Navarro, Maria Navallas, Enrique Ladera, Ferran Torner, Mariona Sunol, Moira Garraus, Jordi Català March, Ignasi Barber

**Affiliations:** 1 Department of Diagnostic Imaging. Hospital Sant Joan de Déu, Av. Sant Joan de Déu, 2, CP:08950 Esplugues de Llobregat, Barcelona, Spain; 2 Department of Medical Imaging, Department of Diagnostic Imaging, The Hospital for Sick Children, University of Toronto, 555 University Avenue, Toronto, ON M5G 1X8 Canada; 3 Department of Diagnostic Imaging, Hospital 12 de Octubre, Madrid, Spain; 4 Department of Orthopaedics, Hospital Sant Joan de Déu. Av, Sant Joan de Déu, 2, CP:08950 Esplugues de Llobregat, Barcelona, Spain; 5 Department of Pathology, Hospital Sant Joan de Déu, Esplugues de Llobregat, Barcelona, Spain; 6 Department of Oncology, Hospital Sant Joan de Déu. Av, Sant Joan de Déu, 2, CP:08950 Esplugues de Llobregat, Barcelona, Spain; 7 Department of Radiology, Instituto de Resonancia Magnetica Guirado, C/Muntaner, 531, CP:08022 Barcelona, Spain

**Keywords:** Ewing sarcoma, MRI, Osteosarcoma, Pediatric

## Abstract

Osteosarcoma and Ewing sarcoma are the most common bone sarcomas in children. Their clinical presentation is very variable depending on the age of the patient and tumor location. MRI is the modality of choice to assess these bone sarcomas and has an important function at diagnosis and also for monitoring recurrence or tumor response. Anatomic sequences include T1- and T2-weighted images and provide morphological assessment that is crucial to localize the tumor and describe anatomical boundaries. Multiparametric MRI provides functional information that helps in the assessment of tumor response to therapy by using different imaging sequences and biomarkers. This review manuscript illustrates the role of MRI in osteosarcoma and Ewing sarcoma in the pediatric population, with emphasis on a functional perspective, highlighting the use of diffusion-weighted imaging and dynamic contrast-enhanced MRI at diagnosis, and during and after treatment.

## Key points

- Volumetric determination of tumor ADC reflects lesion heterogeneity better than cross-sectional analysis.
- Treatment response is based on the evaluation of ADC histograms of the tumor.
- Time intensity curves, together with DWI and conventional sequences, help in the characterization of bone sarcomas.

## Introduction

Bone sarcomas are infrequent neoplasms with an annual incidence of 8–9 cases per million children, accounting for up to 6% of all pediatric malignant tumors [1, 2]. However, they comprise the majority of primary bone tumors in children.

Osteosarcoma and Ewing sarcoma usually have a varied clinical symptoms and radiological presentation depending on two factors: age and location of the tumor within the bone. Radiography plays a paramount role for the initial workup and diagnosis of bone tumors due to its sensitivity to detect bone matrix. Classical signs related to malignancy on conventional radiography include “sunburst periosteal reaction”, “onion skin periosteal reaction”, “Codman triangle” and “permeative pattern” [3, 4]. Radiography should be followed with cross-sectional imaging for initial evaluation of the tumor and also for treatment response to therapy.

MRI is the preferred imaging technique for the study of bone sarcomas, which is accomplished by using anatomic and functional MRI techniques [5]. Anatomic MRI sequences provide anatomic information for accurate assessment of the local disease including extent of intraosseous involvement, soft tissue component, neurovascular bundle involvement, skip lesions and lymphadenopathy [6]. Nonetheless, anatomic sequences remain inadequate to monitor residual and recurrent disease after treatment.

Traditionally, tumor response assessment in pediatric bone sarcomas has been performed using three-dimensional measurements as defined by the ESMO-PaedCan-EURACAN Clinical Practice Guidelines [7] and more recently using one-dimensional (1D) measurement based on the Response Evaluation Criteria in Solid Tumors (RECIST 1.1) [8]. Lesion measurements on RECIST 1.1 have to be assessed either on CT or MRI, since radiography, PET and bone scans are only used to confirm the presence of a lesion. However, the (RECIST 1.1) criteria suggest to measure only the soft-tissue component and in one dimension. Measurement of bone marrow tumor is not valid in RECIST 1.1 and cannot be used to assess [8, 9].

Multiparametric or functional MRI provides additional information to that provided by the conventional sequences, including cellularity, vascularity, fat content and metabolism (Table 1). In pediatric bone sarcomas, techniques such as diffusion-weighted imaging (DWI) and dynamic contrast-enhanced MRI (DCE-MRI) have shown to provide valuable information for treatment response assessment.

**Table 1:** Multiparametric MRI techniques and type of information provided

MRI technique	Information
Diffusion-weighted imaging (DWI)	Cellularity
Dynamic contrast-enhanced MRI	Vascularization
Chemical shift	Fat content
Spectroscopy	Metabolism

Multiparametric or functional MRI adds value to the anatomic or conventional MRI. In clinical practice, it is crucial to evaluate conventional and multiparametric MRI techniques, due to the valuable information that they provide for the study of treatment response.

In this article, we review the clinical features and illustrate the conventional and multiparametric MRI features of osteosarcoma and Ewing sarcoma of bone in children, with emphasis on DWI and DCE-MRI.

## Imaging protocol

The imaging protocol used at our institution (Table 2) using a 3-T MRI scanner (Ingenia; Philips Healthcare, Best, Netherlands) combines conventional (anatomic) and functional or multiparametric sequences.

**Table 2:** MRI protocol for the study of bone sarcomas in a 3-T scanner

	Slice number	FOV (mm)	Voxel size (mm)	Slice thickness (mm)	Gap	TR (ms)	TE (ms)	NEX	Flip angle	TA (m:s)	b value
Coronal T2 DIXON	35	429	0.6 × 0.6	3	0	3300	80	1	90	2:34	
Coronal T1 TSE	35	429	0.5 × 0.6	3	0	700	10	1	90	3:03	
Sagittal T2 DIXON	52	300	0.6 × 0.7	1.5	0	3200	60	1	90	2:38	
Axial T2 DIXON	60	120	0.6 × 0.7	3	0	3600	60	1	90	2:48	
Axial T1 TSE	60	120	0.5 × 0.6	3	0	700	10	1	90	2:09	
Axial DWI	60	120	1.1 × 1.1	3	0	9000	78	1	90	5:08	0/500/800
THRIVE T1 TFE	75 (30DYN)	300	1.5 × 1.5	1.5	0	4	2	1	10	5:08	
Sagittal T1 FS + C	52	300	0.8 × 0.8	1.5	0	600	10	1	90	2:19	
Axial T1 FS + C	60	120	0.5 × 0.6	3	0	700	10	1	90	3:28	

*30DYN* = 30 series after contrast administration. *C* = contrast agent. *DWI* = diffusion-weighted imaging. *FFE* = fast field echo. *FOV* = field of view. *FS* = fat suppression. *m:s* = minutes:seconds. *ms* = milliseconds. *NEX* = number of excitations. *TA* = acquisition time. *TE* = echo time. *TR* = repetition time. *TSE* = turbo spin echo

Multiplanar images are acquired using T1-weighted spin-echo sequence (TR, 700 ms; TE, 10 ms) and T2-weighted fast spin-echo sequence (TR, 3300 ms; TE, 80 ms). For fat-suppressed images, DIXON is preferred as it provides more homogeneous signal intensity (TR, 3200 ms; TE 60 ms), although STIR or fat-suppressed T2-weighted images are routinely used in institutions where it is not available. All images are obtained with a slice thickness of 3 mm with 0-mm interslice gap [2, 4, 10].

DWI is routinely performed in the axial plane using single-shot or multishot spin-echo echo-planar imaging sequences (TE 9000/TR 78 ms; 3-mm slice thickness; and 0-mm interslice gap) and b-values of 0, 500 and 800 s/mm^2^ (Table 2). Factors such as field of view and matrix may vary depending on the region of interest and age of the patient [5].

DCE-MRI evaluation is performed using a THRIVE sequence (T1-weighted high-resolution isotropic volume excitation, fast gradient, 3D and fat saturation) with the following parameters: TR 4 ms; TE 2 ms; slice thickness 1.5 mm; 0-mm interslice gap.

The evaluation of bone sarcomas in children at diagnosis and also during and after treatment requires a detailed MRI assessment, which includes anatomic and multiparametric MRI techniques (DWI and dynamic MRI study), because the imaging results may affect the patient management (Table 3).

**Table 3:** Qualitative MRI features of biological processes involved in bone sarcomas

Biological process	MRI sequences				
	T2	T1 FS + C	DWI	ADC	DCE-MRI (curve profile)
Viable tumor	Isointense	Hyperintense	Increased	Decreased	Types III and IV
Peritumoral edema	Hyperintense	Hyperintense	Increased	Increased	Types II and V
Fibrosis	Hypointense	Hypointense	Increased	Increased	Types II and V
Necrosis	Hyperintense	Hypointense	Increased	Increased	Type I

*ADC* = apparent diffusion coefficient. *C* = contrast agent. *DCE* = dynamic contrast-enhanced. *DWI* = diffusion-weighted imaging. *FS* = fat-suppressed

## Conventional MRI

Anatomic T1-weighted and T2-weighted or STIR MR images provide relevant information for diagnosis, staging, and surgical planning [2, 10].

Bone sarcomas are usually iso- to hypointense to muscle on T1-weighted images, and heterogeneously hyperintense on T2 DIXON or STIR images, due to the presence of hemorrhage and necrosis [2, 6, 9, 10].

On T1-weighted images, osteosarcoma usually shows a sharp transition from normal fatty marrow to hypointense tumor marrow involvement. In contrast, Ewing sarcoma shows a wide intramedullary transition between the tumor and the normal bone marrow [2]. Furthermore, Ewing sarcoma is associated with a soft-tissue mass in 96% of cases [11, 12], while osteosarcoma tends to expand the medullary cavity with bone formation.

MRI of osteosarcoma and Ewing sarcoma should include the primary tumor site, the entire bone in which the tumor is located and the two adjacent joints (proximal and distal to the affected bone) in order to identify any potential skip lesion [6]. This is usually achieved by imaging in the coronal plane on T1 without fat suppression and on fat-suppressed T2 using a body coil for the initial evaluation. Then, a dedicated examination of the tumor is performed with a surface coil. T1-weighted images are very helpful to delineate the boundaries of the bone marrow involvement, whereas gradient echo sequences are not sufficiently accurate. The length of the tumor is usually measured on T1-weighted images from the articular surface to the point at which marrow signal intensity changes from abnormal to normal. This information is helpful for planning the construction of the individual prosthesis for the patient. Longitudinal measurement is routinely performed on T1-weighted images because DIXON or STIR sequences may significantly overestimate the bone marrow involvement, as edema and marrow hyperplasia show high signal intensity similar to that of tumor [10].

## Multiparametric MRI

### Diffusion-weighted imaging

Diffusion-weighted imaging (DWI) is a functional MRI technique based on the random Brownian motion of water molecules within a voxel of tissue. The apparent diffusion coefficient (ADC) is a quantitative method to evaluate tumor cellularity, which can be of substantial importance when assessing treatment response: high cellular tumors are represented by low ADC values, whereas lower cellular tumors are represented by high ADC values. As a consequence, DWI provides quantitative functional evaluation of the cellularity, allowing the potential differentiation between benign and malignant lesions as well as improvement of the MRI assessment of treatment response [5, 13, 14].

The viability of the tumor can be characterized by longitudinally monitoring changes in ADC tumor heterogeneity at different stages of treatment. Both necrotic and solid parts of the tumor need to be routinely included in the sample to facilitate reproducibility of this method [14, 15].

The ADC values are dependent on the MR system including the vendor, magnetic field strength and sequence parameters and usually cannot be directly translated to other MR scanners. However, ADC can be calibrated between different MR systems using phantoms in order to ensure some comparability [16].

There are different methods to measure ADC values. ADC is usually acquired by drawing a region of interest (ROI) in the most cellular area of the tumor, and this may provide information about the mean value or ADCmean, minimal ADC value or ADCmin and the maximal ADC value or ADCmax. ADCmin represents the most cellular component of the tumor [17]. ADCmean and ADCmax include non-viable necrotic and cystic portions of the tumor. ADCmean represents the mean tissue composition and has been correlated with proliferation potential of the tumor, while ADCmax has been associated with low cellular component of the tumor [18–23].

Volumetric determination of tumor ADC (ADC histogram) shows better the lesion heterogeneity, compared with a single, cross-sectional lesion region of interest (ROI) analysis [15, 16]. ADC histogram using a volumetric determination of the lesion analysis is useful to evaluate treatment response in soft-tissue tumors and also in patients with malignant bone tumors [15–17]. Moreover, ADC volumetry applied only on areas of high cellularity that correlates with restriction of the tumor after chemotherapy may be used as a way to estimate the volume of viable tumor [24, 25].

ADC histogram is a valuable tool to assess response to treatment in patients with bone sarcomas. Hence, it needs to be performed not only at diagnosis but also during and after completion of the treatment in order to evaluate treatment response. Therapeutic response correlates with the degree of movement of the histogram through the horizontal axis to the right. In the event of significant response to treatment, ADC values of the tumor tend to increase and the histogram displaces to the right of the graph. However, in the event of no therapeutic response to treatment, there is usually overlap between histograms or no movement of the curve through the horizontal axis. [23–27].

Intravoxel incoherent motion (IVIM) is based on the acquisition of DWI with b-values below 200 s/mm^2^. Thus, the images become sensitive to the motion of water molecules in the intravascular space (i.e., perfusion) in addition to water molecules diffusing in the extracellular and intracellular compartments [28]. Quantitative IVIM analysis of DWI signal may distinguish the perfusion component from tissue diffusion and this may help in better tissue characterization and treatment monitoring [28].

### Perfusion MRI

Perfusion MRI can be performed using unenhanced sequences such as DWI, or with enhanced sequences such as DCE-MRI, which requires the use of contrast material [29, 30].

DCE-MRI relies on the use of TRICKS or TRAK sequences that are characterized by a low temporal resolution acquisition usually less than 6 s for 5 min [29]. Analysis of DCE-MRI sequence can be done using various postprocessing techniques, in a quantitative or qualitative manner.

Quantitative assessment of DCE-MRI allows the measurement of tissue microvasculature properties. A meta-analysis performed by Kubo et al. [31] showed that the curve slope derived from DCE-MRI is useful for the assessment of treatment response after chemotherapy in osteosarcoma and in Ewing sarcoma.

DCE-MRI measures the properties of tissue microvasculature using contrast material. The first pass of the contrast bolus provides information about blood flow and volume, and this is calculated according to the signal variation over the first pass of the contrast. The accumulation of contrast agent in the tissues and its elimination is estimated over longer time frames [31, 32].

A qualitative analysis approach relies on time-signal intensity curves (TIC) or curve profile. The study of the TIC morphology provides information about the biological process of the tumor and allows the characterization of the tumor before and after treatment [33–35].

**Fig. 1:**
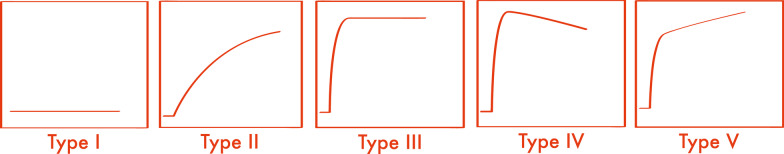
Time intensity curves (TIC) in dynamic contrast-enhanced MRI. Type I profile represents necrosis or cystic changes; type II is associated with benignity, such as peritumoral edema; types III and IV with malignant processes; and type V with granulation tissue or fibrosis

The use of TIC derived from DCE-MR has been widely reported in the literature in different clinical scenarios and parts of the body. According to Lavini et al. [36], there are 5 types of curve profile (Fig. 1). Type 1 curve shows no contrast uptake resulting in a horizontal type curve; type 2 shows progressive enhancement; type 3 shows a delayed plateau; type 4 shows a delayed washout; and type 5 shows fast initial contrast uptake followed by progressive late enhancement. Type 1 profile correlates with necrosis, type 2 with benign processes, such as peritumoral edema, types 3 and 4 with malignancy, and type 5 with granulation tissue or fibrosis [36–38]. Lavini et al. also describe two other curves. Type 6 or artery, which is characterized by a quick uptake and quick wash-out, and type 7, which includes all others curves not classified in the previous types.

**Fig. 2:**
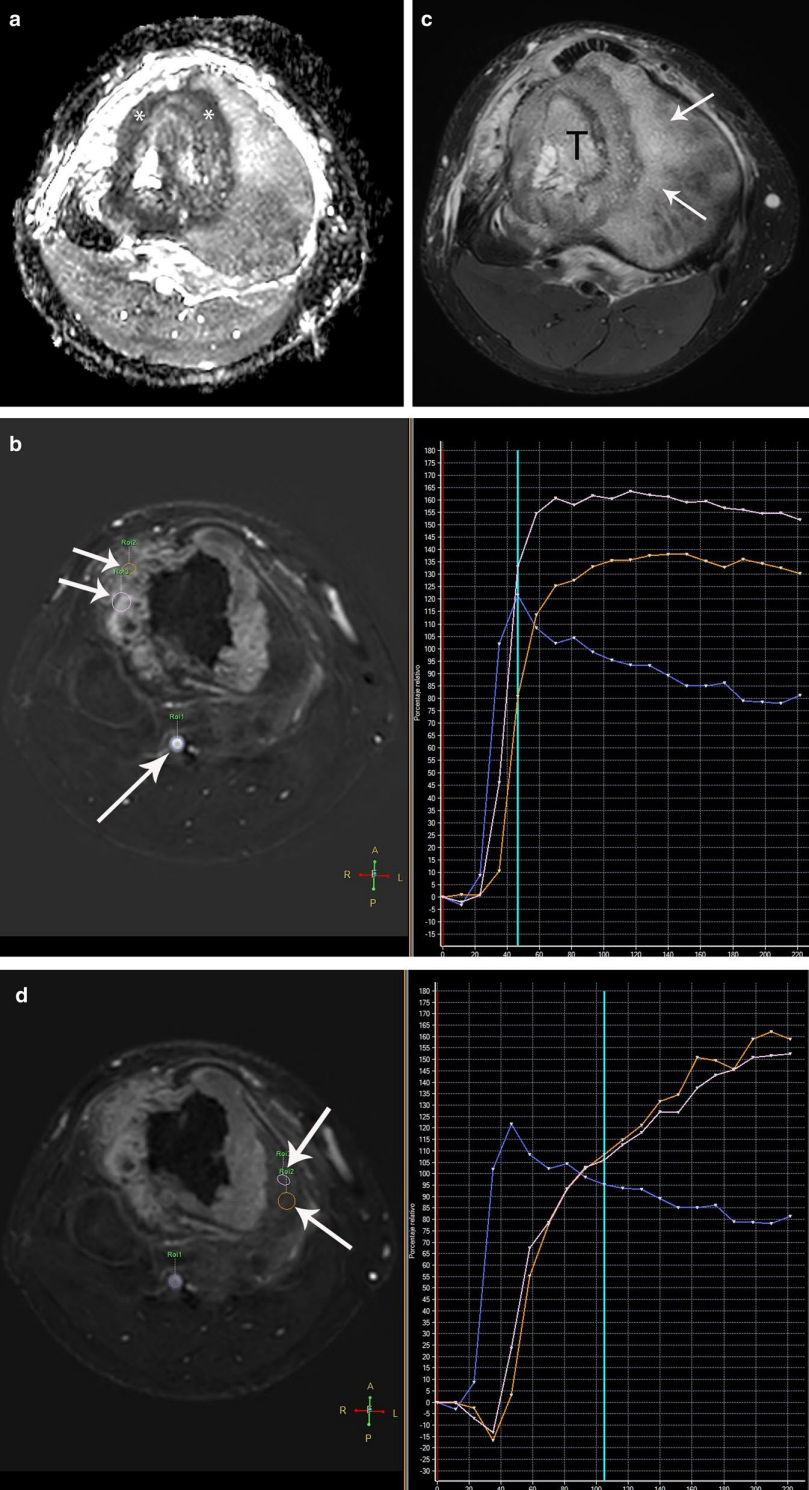

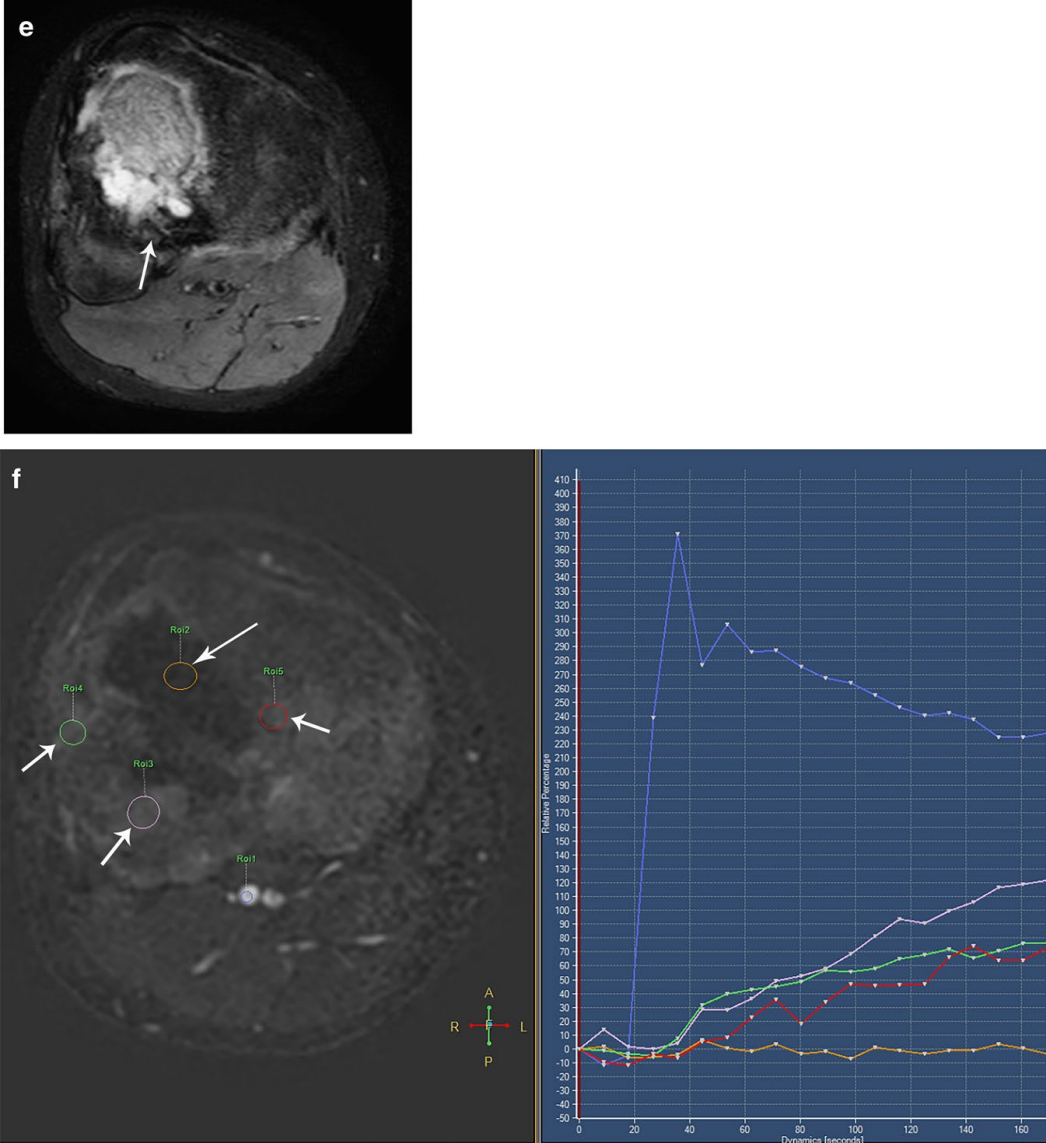
Osteosarcoma of the proximal right tibia in a 16-year-old boy. **a** Axial ADC map shows low signal intensity of the tumor confirming restricted diffusion (*). **b** Axial DCE-MR image (left) with corresponding time intensity curve (TIC) (right) shows peripheral enhancement of the tumor (short arrows on the left) which correlates with a type III curve (pink and orange arrows on the TIC). The blue curve in the TIC shows the typical morphology of any peripheral artery (long arrow on the left). **c** Axial fat-suppressed T2-weighted MR image shows hyperintensity of the bone marrow (arrows) surrounding the tumor (T), in keeping with edema. **d** Axial DCE-MR image (left) and corresponding TIC (right) show progressive enhancement of the bone marrow (arrows) which correlates with a type II curve (orange and pink curves), indicating edema. The blue curve in the TIC shows the typical morphology of the peripheral artery. **e** Axial fat-suppressed T2-weighted MR image after 2 cycles of chemotherapy shows an area of predominantly low signal intensity within the tumor (arrow) that may represent fibrosis and/or calcification. **f** Axial DCE-MR image (left) with corresponding TIC (right) shows progressive enhancement of the peripheral area of the tumor (short arrows) with a type II curve (red, green and pink curves), indicating overall good treatment response and presence of granulation tissue. Note the lack of enhancement of the central part of the tumor (long arrow) due to necrosis that correlates with a type I curve (orange curve) indicating non-viable tumor or necrosis. The blue curve correlates with the typical morphology of a peripheral artery

Dynamic MRI study may differentiate areas of necrosis, viable tumor, fibrosis and peritumoral edema, because they show specific types of TIC (Fig. 2). Although there may be overlap, DCE-MRI can help differentiate a malignant tumor from a benign tumor or at least narrow the differential diagnosis [39].

Areas of viable tumor are isointense on T2-weighted images, heterogeneously hyperintense following contrast administration and show low ADC values corresponding with high cellular areas. On DCE-MRI, viable tumor shows avid arterial contrast enhancement corresponding with higher slopes and type III and IV TIC.

Fibrosis and peritumoral edema usually share the same MRI features. On T2-weighted and contrast-enhanced fat-suppressed T1-weighted images, they show hyperintensity with high ADC values reflecting low or absent cellular areas. On DCE-MRI, the enhancement may be rapid, sustained and progressive or in contrast there may be progressive enhancement, but in any case they are associated with type II and V TIC.

Necrosis is usually hyperintense on T2-weighted images with no enhancement after contrast administration and shows high ADC values reflecting low cellularity. As there is no contrast uptake, DCE-MRI shows no curve slope correlating with type I TIC [39].

Osteosarcoma and Ewing sarcoma of bone usually show early arterial enhancement, which needs to be correlated and evaluated with DWI and conventional MRI.

The TIC shape analysis does not provide absolute measurements, and it is partly dependent on the protocol chosen. The final shape of the curve may be affected by several factors, such as the length of the dynamic MRI study, time resolution, and scan features [37, 38].

There are some limitations regarding the use of DCE-MRI. Type III TIC and type IV TIC are described in almost all patients with high-grade bone sarcomas and metastases. However, considering that the slope of the curve is partly affected by the vascularization of the tumor, poorly vascularized malignant tumors may exhibit benign slopes, whereas highly vascularized benign tumors, such as giant cell tumor and eosinophilic granuloma, may falsely show malignant slopes [33, 35].

Therefore, the rapid arterial uptake on DCE-MR is a better indicator of cellularity, cell turnover time, and vascularization, rather than the potential malignancy of bone tumors.

Osteosarcomas are usually highly perfused and permeable, while after chemotherapy, the tumors show substantial decrease in both perfusion and permeability [34, 39]. This corresponds to either development of tumor necrosis with fibrosis or to regions of relatively dormant tumor. Areas of persistent enhancement imply that some tumor has not responded effectively to therapy [32, 34]. The acquisition of delayed contrast-enhanced fat-suppressed T1-weighted images after completion of the dynamic study is useful because this improves visualization of the margins of the tumor and of the involvement of the neurovascular bundle and allows a more accurate estimation of tumor necrosis.

## Osteosarcoma

### Clinical presentation

Osteosarcoma is the most common primary malignant bone tumor, often occurring in adolescents between 12 and 16 years of age, with a second peak incidence in patients older than 60 years [40]. Conventional type is the most common in childhood and adolescence and has been divided according to histologic appearance into osteoblastic, chondroblastic, and fibroblastic [41].

Children usually present with regional pain and occasionally in combination with a palpable mass or swelling [40]. Some patients relate the onset of symptoms to trauma occurring at the time that the symptoms began. Some bone tumors may present with neurologic symptoms depending on the site of origin [41, 42].

### Conventional MRI findings

Osteosarcoma often affects the metaphysis and diaphysis of long bones [4]. It is iso- to hypointense to muscle on T1-weighted images, hyperintense on T2-weighted images, and shows heterogeneous enhancement after contrast agent administration [2]. As the tumor grows, MRI shows associated soft-tissue mass. Osteosarcoma not only metastasizes to the lungs, but also spreads within the affected bone resulting in skip lesions.

### Multiparametric MRI findings

**Fig. 3:**
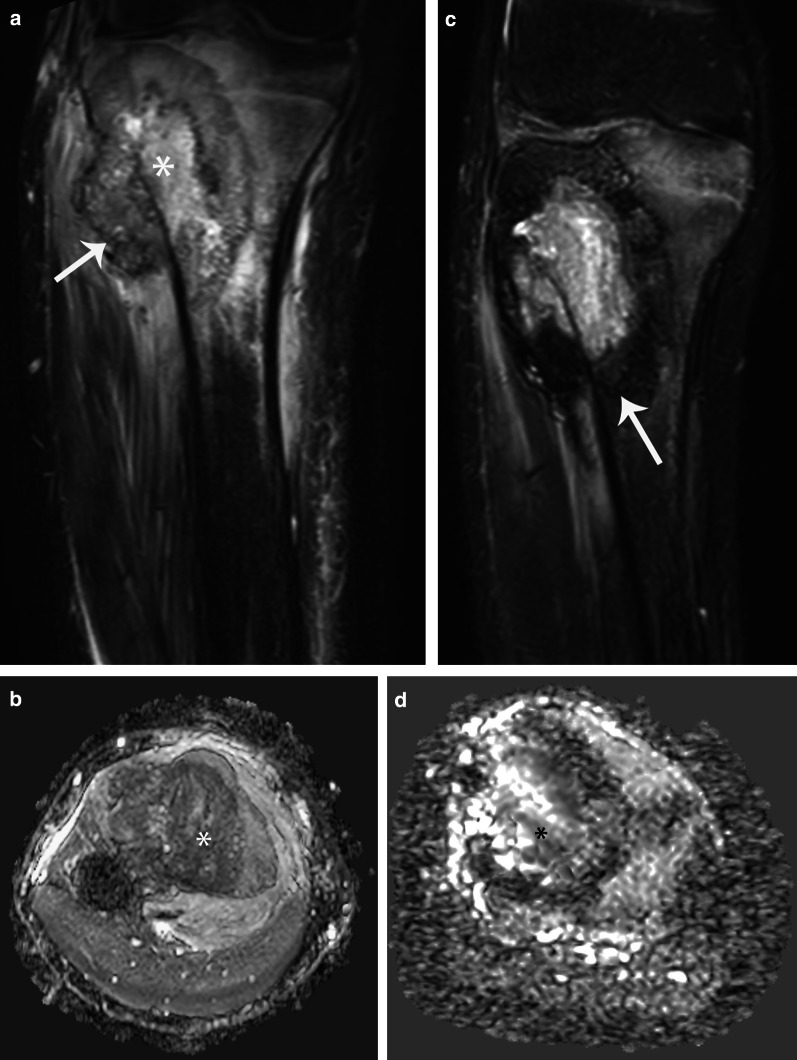
Osteosarcoma involving the proximal metaphysis of the tibia in a 13-year-old boy. **a** Coronal fat-suppressed T2-weighted MR image shows a heterogeneous bone lesion (*) associated with a soft-tissue mass (arrow). **b** Axial ADC MR image shows diffusion restriction within the tumor (*). **c, d** Post-chemotherapy MRI. **c** Coronal fat-suppressed T2-weighted MR image shows a peripheral hypointense rim in the tumor suggestive of calcification (arrow). **d** Axial ADC MR image shows decrease of the areas of diffusion restriction (*) compared to the initial study, indicating good treatment response

Osteosarcoma shows restricted diffusion due to its high cellularity, and this correlates with low ADC values [13–15, 18] (Fig. 3). Treatment response after chemotherapy with DWI should be analyzed with caution depending on the histological subtype of osteosarcoma. ADC values tend to be significantly different between viable tumor and necrosis in fibroblastic and osteoblastic osteosarcoma, whereas viable chondroblastic osteosarcoma cannot be completely distinguished from necrosis due to its high signal on T2-weighted images and higher ADC values as compared to the conventional type. [43–45]. Moreover, the imaging evaluation of chondroblastic osteosarcoma after neoadjuvant chemotherapy is challenging because cartilaginous tumor survival areas usually show high ADC values, indistinguishable from post-necrotic tissue or cystic/hemorrhagic necrosis [44]. The MRI evaluation of telangiectatic osteosarcoma can also be challenging as it usually has a cystic appearance with fluid levels due to hemorrhage and shows high ADC values.[45, 46].

**Fig. 4:**
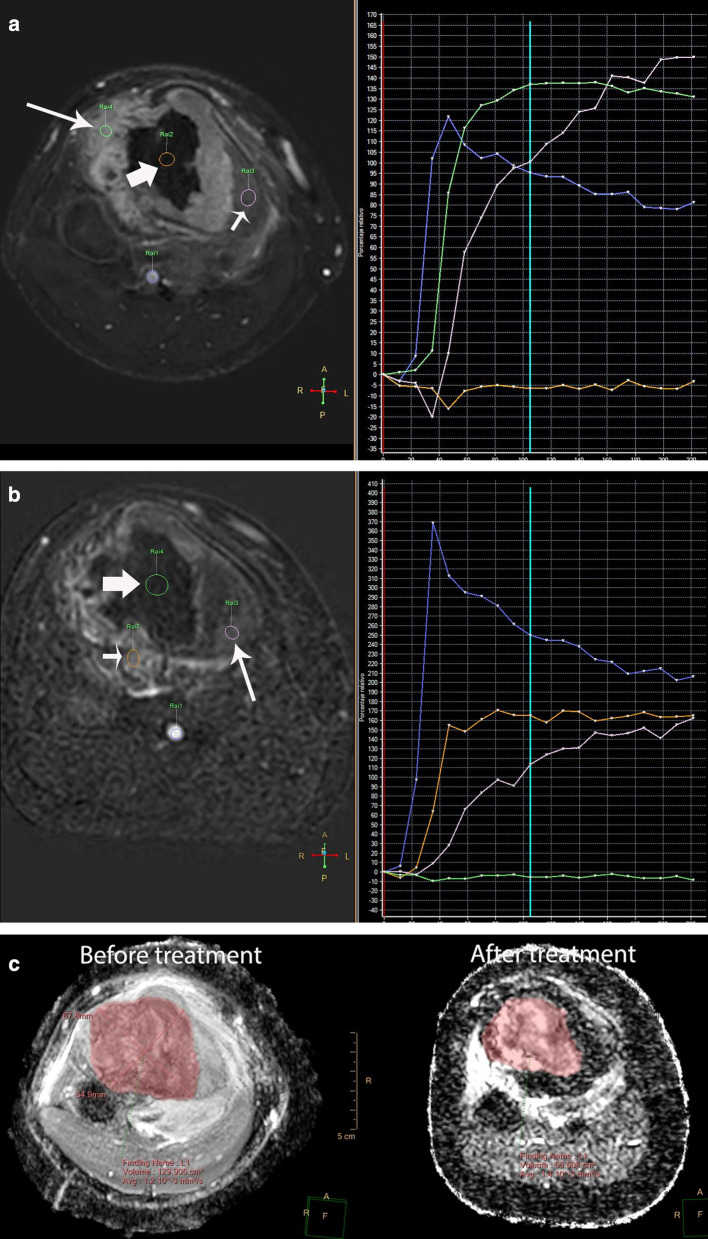

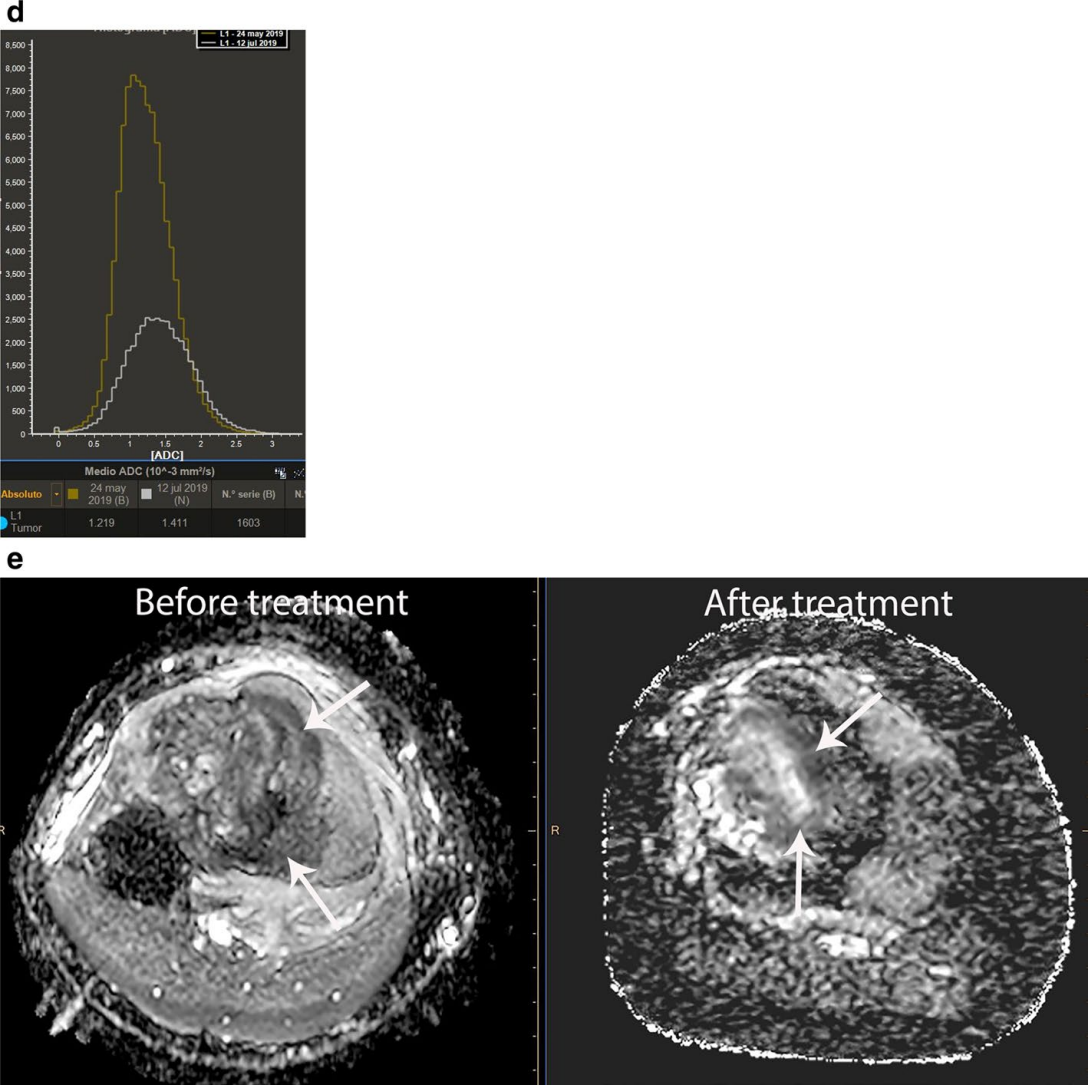
Same patient as in Fig. 3**a** Axial DCE-MR image (left) with corresponding time intensity curve (TIC) (right) before treatment shows early peripheral enhancement of the tumor (long arrows), which correlates with types III and IV TIC (green curve) and no enhancement of the central part of the tumor (thick arrow), which correlates with a type I TIC (horizontal orange curve), suggestive of necrosis. Peritumoral edema (short arrow) correlates with a type II TIC (pink curve). The blue curve represents the arterial TIC. **b** Axial DCE-MR image (left) with corresponding TIC (right) after treatment shows enhancement of the residual tumor (short arrow), which correlates with a type III TIC (orange curve). There is no change of the tumor necrosis (green curve) or peritumoral edema (pink curve). **c** Volumetry and **d** histogram of ADC maps performed before and after treatment shows an increase of the ADC mean values from 1.22 mm^2/^s to 1.41 mm^2/^s. e) ADC image after treatment shows residual tumor (short arrows)

DCE-MRI performed on areas of viable tumor shows types 3 and 4 curve profiles, whereas DCE-MRI performed on areas of non-viable tumor may show either type 1 curve in areas of necrosis or types 2 or 5 curves in areas of peritumoral edema [36–38] (Fig. 4). As with other neoplasms, residual tumor typically shows types 3 and 4 curve profiles, whereas fibrosis or granulation tissue may show type 5 curve profile [36] (Fig. 4).

## Ewing sarcoma of bone

### Clinical presentation

Ewing sarcoma of bone is the second most common primary malignant bone tumor in children and adolescents, accounting for approximately 3% of all pediatric tumors, with an annual incidence of 1–3 cases per 1 million [36, 37]. The age of presentation is very broad, but the mean age of presentation is 14–15 years. There is equal gender distribution [12, 47]. According to the last WHO classification, Ewing sarcoma is included in the group of undifferentiated small round cell sarcomas of bone and soft tissue [48].

The clinical presentation varies depending on the location of the tumor. Symptoms such as pain and mass or swelling are often present for several months before diagnosis. Ewing sarcoma may also simulate an infectious process at presentation, often correlating with advanced disease and metastases [3]. Other manifestations depend on the site of involvement and may include neurologic symptoms, chest pain, and limb length discrepancy.

### Conventional MRI findings

**Fig. 5:**
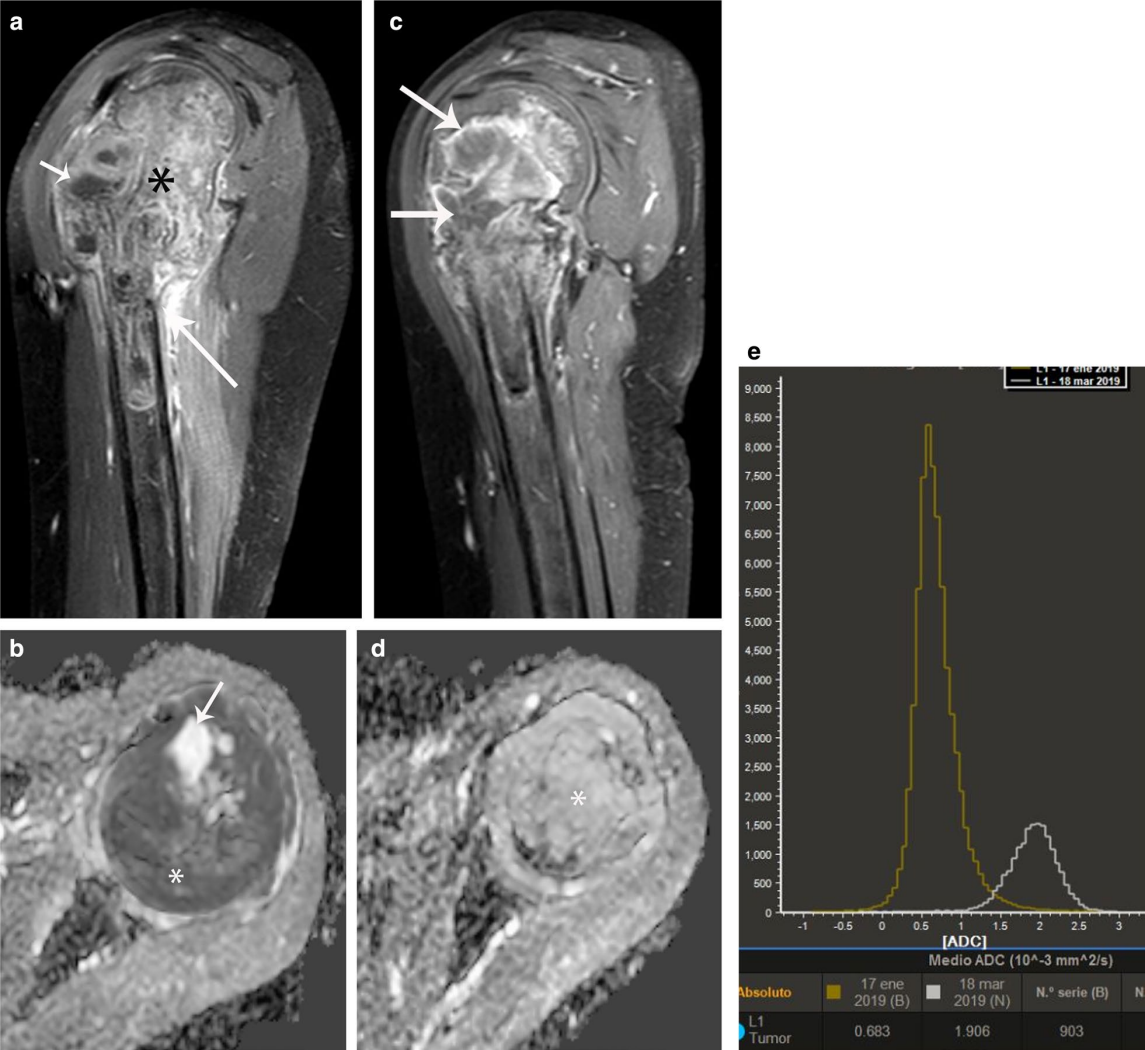
Ewing sarcoma involving the proximal left humerus in a 12-year-old boy. **a** Sagittal contrast-enhanced fat-suppressed T1-weighted MR image shows a heterogeneous mass centered in the proximal humeral metaphysis (*) that extends to the epiphysis and proximal diaphysis and contains foci of internal necrosis (short arrow). Note severe varus angulation of the humerus due to previous fracture (long arrow). **b** Axial ADC MR image shows restricted diffusion of the cellular areas (*) with no restriction of the necrotic foci (arrow). **c** Sagittal contrast-enhanced fat-suppressed T1-weighted MR image after treatment shows decrease in volume of the mass with increase of necrotic areas (arrows). **d** Axial ADC MR image after treatment shows no restricted diffusion (*). **e** Volumetry of ADC maps performed before treatment shows a high and sharp ADC histogram of 0.7 mm^2/^s, while the histogram after treatment is short and wide and shifted to the right, with a value of 1.9 mm^2/^s. The conventional MRI sequences together with DWI indicate good treatment response

Ewing sarcoma usually affects the diaphysis of long bones and presents as a large bone tumor with ill-defined margins associated with a large soft-tissue mass [2] (Fig. 5). It has low to intermediate signal intensity on T1-weighted images and heterogeneous high signal intensity on T2-weighted images. It typically shows inhomogeneous contrast enhancement caused by the presence of internal necrosis or hemorrhage, which, depending on its time of evolution, may appear hyper-, iso-, or hypointense to muscle. Ewing sarcoma typically shows a microcystic appearance on T2-weighted images [2, 12, 48]. Locally, it can be very aggressive, invading surrounding structures.

### Multiparametric MRI findings

**Fig. 6:**
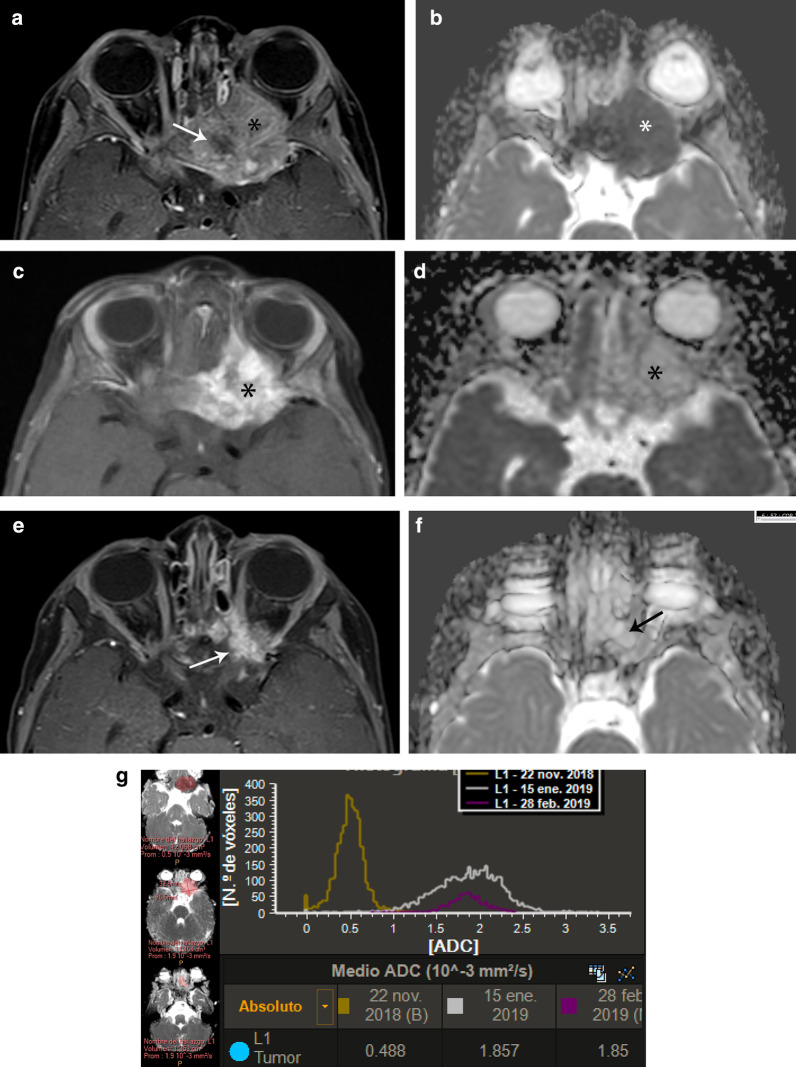
Ewing sarcoma involving the sphenoid bone in a 15-year-old boy. **a** Axial contrast-enhanced fat-suppressed T1-weighted MR image of the orbits shows a heterogeneously hyperintense mass (*) arising from the sphenoid bone with internal necrosis (arrow). **b** Axial ADC map shows restricted diffusion of the mass (*). **c** Axial contrast-enhanced fat-suppressed T1-weighted MR image performed after 2 cycles of chemotherapy shows decrease in volume of the mass with heterogeneous enhancement of the residual tumor (*). **d** Axial ADC maps shows no restriction of the residual mass (*). **e** Axial contrast-enhanced fat-suppressed T1-weighted MR image performed after 3 cycles of chemotherapy shows residual enhancing tissue (arrow). **f** Axial ADC map shows no restriction (arrow). **g** Volumetry of ADC maps performed before (yellow) during (white) and after (purple) treatment. Before chemotherapy, the tumor volume is 12 cc with corresponding high and sharp ADC (yellow) histogram of 0.49 mm^2/^s. During treatment, the volume does not significantly change (11 cc), but the corresponding volumetric ADC (white) histogram increases to 1.85 mm^2/^s, appearing short, wide and shifted to the right. After treatment, the volume decreases to 1.35 cc and the ADC (purple) histogram does not change (1.85 mm^2/^s). Overall, this indicates good treatment response

Ewing sarcoma is a highly cellular tumor and usually shows restricted diffusion, which correlates with low ADC values [2, 5]. The analysis of a combined whole-lesion ADC histogram together with a dynamic MRI assessment before and after treatment, provides a quantitative interpretation of tumor response (Figs. 5 and 6).

According to Parlak et al., absolute ADCmin values have been successfully used to differentiate Ewing sarcoma from osteosarcoma in difficult cases. ADCmin values in Ewing sarcoma have been reported to be significantly lower than in osteosarcoma, being 0.566 ± 0.07 in the former and 1.193 ± 0.33 × 10–3 mm2/s in the latter [49].

TICs are very helpful in the post-chemotherapy setting. Curves performed on areas of viable tumor correlate with types 3 and 4 TIC morphology on dynamic MRI evaluation, whereas TIC performed on areas of non-viable tumor may show type 1 curves indicating necrosis, or type 2 and 5 curves indicating fibrosis or granulation tissue [34–36]. Depending on the location, Ewing sarcoma may be unresectable and the use of dynamic MRI examination and TIC is very useful in order to detect residual disease.

### Clinical applications

**Fig. 7:**
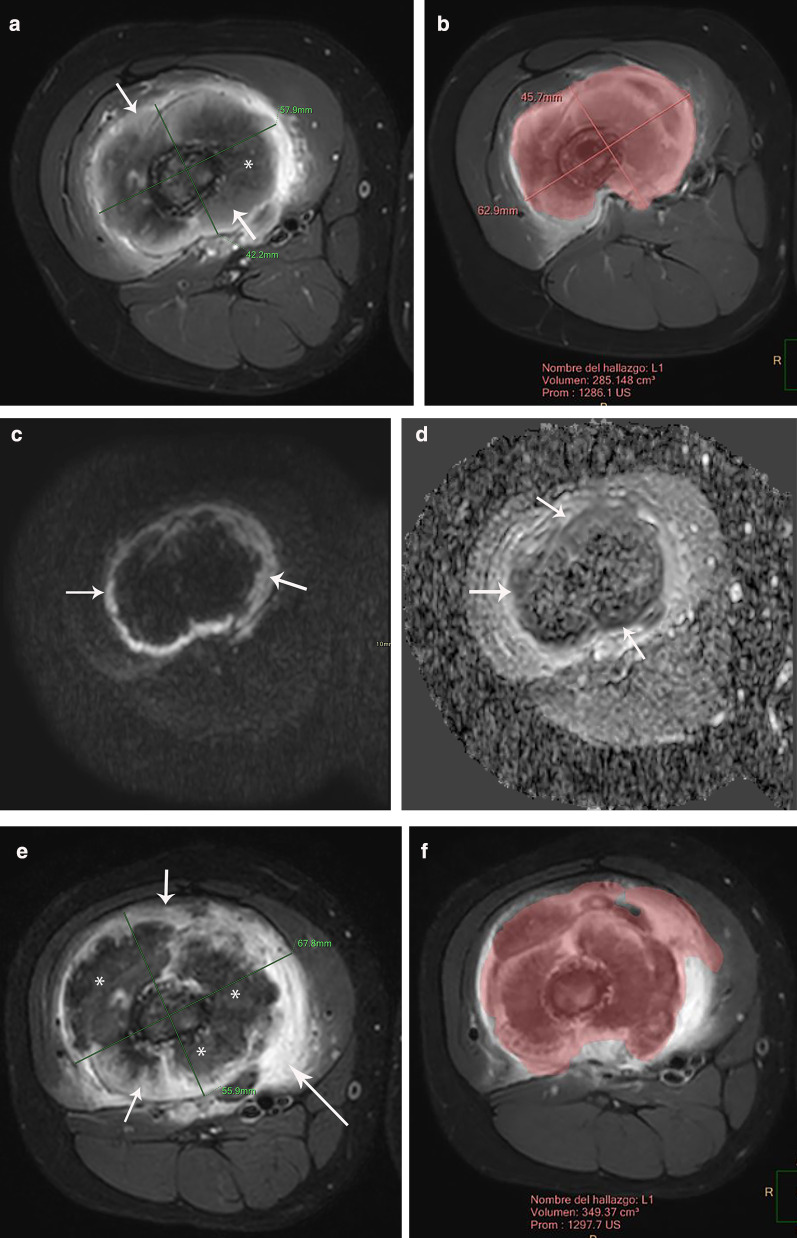

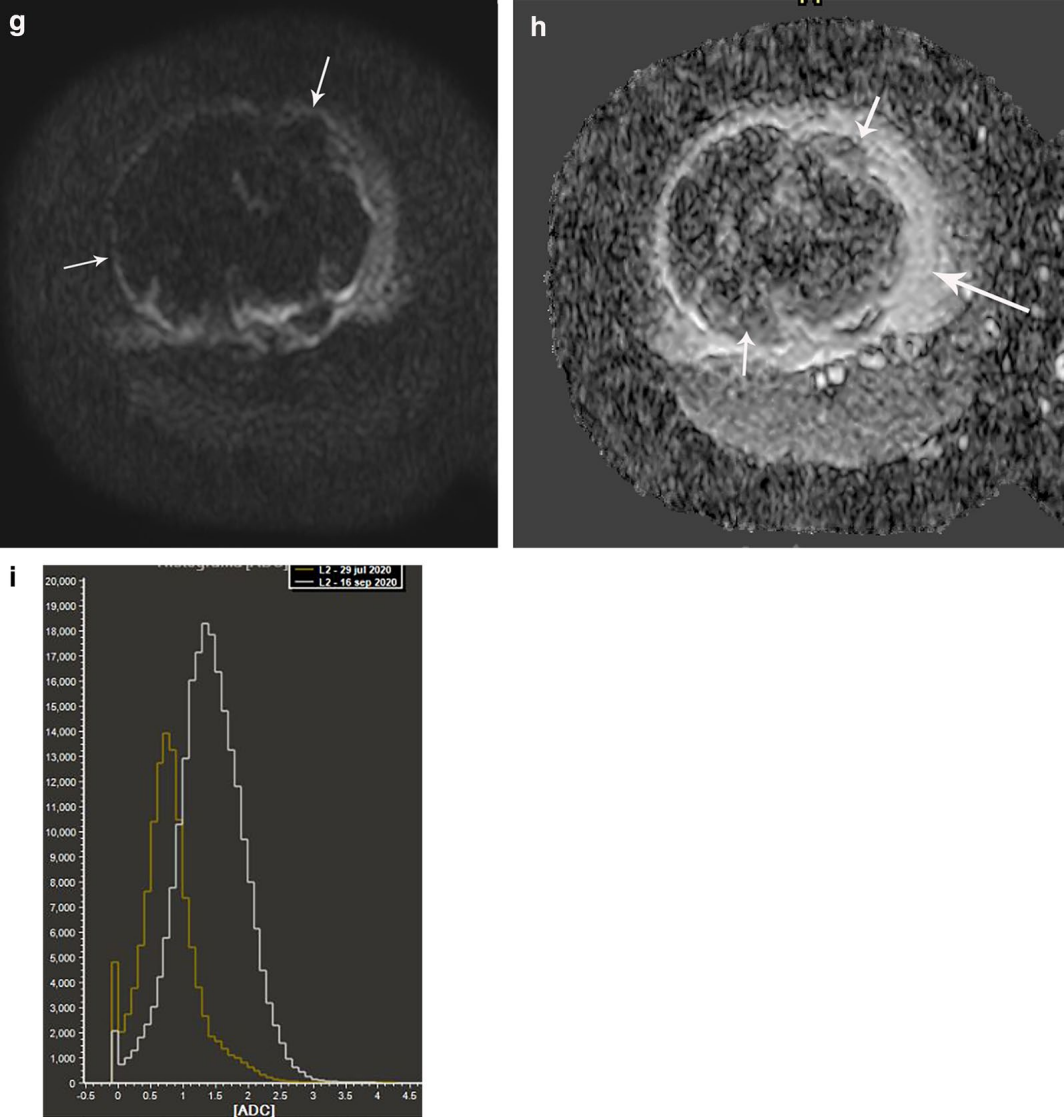
Ewing sarcoma of the distal femur in a 9-year-old boy. a-d) Pre-treatment. **a** Axial contrast-enhanced fat-suppressed T1-weighted MR image before treatment shows a large tumor heterogeneously enhancing (short arrows) with internal necrosis (*). **b** Automated volumetry of the mass is around 285 cc. **c** and **d** Axial DWI and ADC map images show restricted diffusion in the periphery of the lesion (arrows). Post-treatment. **e** Axial contrast-enhanced fat-suppressed T1-weighted MR image after treatment shows increase of the internal necrosis of the mass (*) with persisting peripheral enhancement (short arrows). Note soft tissue inflammation not present in the previous MRI (long arrow). **f** Automated volumetry of the mass is around 350 cc. **g** and **h** Axial DWI and ADC map images show mild linear restricted diffusion of the periphery (arrows). **i** Histogram of ADC maps performed before and after treatment shows an increase of the ADC mean values from 0.79 mm2/s to 1.41 mm2/s

The use of neoadjuvant chemotherapy in bone sarcomas causes changes in the tumor that may be difficult to interpret by only relying on conventional MRI. For example, the treatment may produce changes of the intralesional signal intensity as well as increase in size due to necrosis and inflammatory events. The interpretation of this new appearance of the tumor on conventional MRI is challenging and difficult to differentiate from residual tumor [31, 32, 36] (Fig. 7). In these cases, the use of multiparametric MRI may be helpful to differentiate viable tumor from necrosis and granulation tissue [5, 34, 36, 37]. Multiparametric MRI should always be analyzed in conjunction with the anatomic sequences to avoid misinterpretations.

Further improvement of the MRI techniques and sequences will allow radiologists a better interpretation of the studies and the potential role of DCE-MRI and DWI in determining the better timing to use neoadjuvant therapy [22, 31, 38]. Habre et al. demonstrated that ADC values are very helpful to evaluate response to therapy at mid-course of chemotherapy in bone sarcomas to differentiate good responders from bad responders. The change of absolute mean ADC values before and after chemotherapy is usually significantly higher among the good responders compared to the poor responders. For this reason, DWI is an excellent imaging technique to evaluate early treatment response as well as bad or no response, both of which could have clinical impact when monitoring young patients with bone sarcomas [50].

Machine learning applied to multiparametric MRI may provide in the near future an objective and reliable method to assess response to neoadjuvant chemotherapy in bone sarcomas. Huang et al. proved that machine learning methods together with the information provided by anatomic (T2-weighted and post-contrast images) and functional (ADC) MRI might confidently discriminate between tumor necrosis from tumor survival [51].

## Conclusion

The combination of conventional and multiparametric MRI is essential in the evaluation of osteosarcoma and Ewing sarcoma in children as it provides valuable data on the detection, characterization, staging, and follow-up after treatment. Quantification of tumor ADC heterogeneity has the potential to serve as a marker of treatment response and may be predictive of a tumor's potential response to therapy.

DWI characteristics and enhancement patterns are different for viable tumor, granulation tissue, peritumoral edema and necrosis. Bone sarcomas usually show restricted DWI and types 3 and 4 curve profiles. Necrosis usually shows a type 1 curve profile. Fibrosis, granulation tissue and peritumoral edema usually show types 2 or 5 curve profiles.

## Data Availability

Not applicable.

## Abbreviations

ADC: Apparent diffusion coefficient
DCE-MRI: Dynamic contrast-enhanced MRI
DWI: Diffusion-weighted imaging
ESMO-PaedCan-EURACAN: European society for medical oncology-paediatric cancer-European reference on rare adult solid cancer
IVIM: Intravoxel incoherent motion
MRI: Magnetic resonance imaging
RECIST: Response evaluation criteria in solid tumors
ROI: Region of interest
STIR: Short tau inversion recovery
TE: Time of echo
THRIVE: T1-weighted high-resolution isotropic volume excitation
TIC: Time-signal intensity curve
TR: Time of repetition
TRAK: Time-resolved angiography with keyhole
TRICKS: Time-resolved imaging of contrast kinetics
